# The Transcription Factor C-Myc Suppresses MiR-23b and MiR-27b Transcription during Fetal Distress and Increases the Sensitivity of Neurons to Hypoxia-Induced Apoptosis

**DOI:** 10.1371/journal.pone.0120217

**Published:** 2015-03-17

**Authors:** Qun Chen, Fan Zhang, Yanbo Wang, Zhengya Liu, Anyang Sun, Ke Zen, Chen-yu Zhang, Qipeng Zhang

**Affiliations:** 1 State Key Laboratory of Pharmaceutical Biotechnology, Jiangsu Engineering Research Center for microRNA Biology and Biotechnology, School of Life Sciences, Nanjing University, Nanjing, China; 2 Laboratory of Neurodegenerative Diseases and Repair, Yancheng Institute of Health Sciences, Yancheng, China; Wayne State University, UNITED STATES

## Abstract

Previous studies reported that the expression of miR-23b-27b cluster was downregulated in embryonic brain cortices during hypoxia-induced neuronal apoptosis. However, the mechanism underlying this downregulation is not completely understood. Here, we report that the transcription factor c-Myc plays an important role in regulating the expression of miR-23b-27b cluster during hypoxia. First, the c-Myc protein level was significantly elevated in embryonic brain cortices in a mouse model of fetal distress. Second, forced overexpression or knockdown of c-Myc could suppress or increase the expression of miR-23b-27b cluster polynucleotides. Third, we identified 2 conserved c-Myc binding sites (E-boxes) in the enhancer and promoter regions of miR-23b-27b cluster in the mouse genome. Finally, we showed that elevated c-Myc expression led to an increase in the Apaf-1 level by suppressing miR-23b-27b cluster expression and that this enhanced neuronal sensitivity to apoptosis. In summary, our study demonstrates that c-Myc may suppress the expression of the miR-23b-27b cluster, resulting in additional neuronal apoptosis during hypoxia.

## Introduction

Fetal distress, which includes defects in the fetus due to low oxygen levels during the antepartum or intrapartum periods, often leads to acquired neonatal brain damage. Depending on the severity and duration of the insult, the resulting neuro-dysfunction can involve the entire central nervous system [1]. Hypoxia-ischemia leads to different neuropathological manifestations in premature and term infants. Neuronal injury predominates in term infants, whereas oligodendroglial/white matter injury predominates in premature infants [2]. Deficits in learning and memory, hippocampal plasticity and motor function have been observed in the absence of gross hippocampal cell death in a model of near-term birth asphyxia in the spiny mouse [3–5]. Hypoxic cerebral damage mainly results in neuronal loss via necrosis or apoptosis [6]. Hypoxia is also an important factor in the pathogenesis of several other diseases such as stroke and cancer [7]. Several lines of evidence indicate that hypoxia induces cell death through the apoptotic protease activating factor-1 (Apaf-1)-mediated mitochondrial pathway and results in the activation of caspase-3 [6–8]. Apaf-1 has been reported to be upregulated under hypoxic conditions in the cerebral cortex of newborn piglets [9] and of fetal mice [10]. Hypoxia—ischemia can increase the levels of both Apaf-1 and cytochrome c in the neonatal rat forebrain [11]. We also reported that the protein level of Apaf-1 in neurons is upregulated during hypoxia and that this upregulation of Apaf-1 may be regulated by microRNAs at the post-transcriptional level [10].

miRNAs (microRNAs) regulate gene expression at the post-transcriptional level by binding to the 3’-untranslated regions of target mRNAs to either block mRNA translation or trigger mRNA degradation [12–14]. Thus, miRNAs can regulate diverse cellular functions and play important roles in a wide variety of physiological and pathological cellular processes [15]. In nervous system diseases, hypoxia triggers aberrant expression of miRNAs in neural [16,17] as well as nasopharyngeal cells [18]. Our previous study showed that miR-23b and miR-27b in neurons, both of which are encoded by the miR-23b-27b-24–1 cluster, are downregulated during hypoxia, resulting in the upregulation of Apaf-1 and the promotion of apoptosis both *in vitro* and *in vivo* [10]. However, the mechanism by which miR-23b and miR-27b are downregulated by hypoxia has not been investigated.

The oncogene c-Myc encodes a conserved basic helix-loop-helix leucine zipper transcription factor and is known to regulate miRNA expression at the transcriptional level by binding to a conserved E box (CACGTG) [19]. Cells overexpressing c-Myc have been shown to be more sensitive to the induction of apoptosis under stress [20,21]. In particular, Greenway et al reported that the effect of hypoxia on c-Myc in turtles is organ-specific, and c-Myc protein expression is increased in kidney and brain after hypoxia [22]. Hung et al reported that chemical hypoxia causes 2.2-fold increases in c-Myc mRNA expression in a rat brain-derived type 2 astrocyte cell line [23]. It has been reported that c-Myc represses miR-23a and miR-23b at the transcriptional level in human P-493B lymphoma cells and PC3 prostate cancer cells [24]. More recently, Li et al successfully repeated this experiment and found that c-Myc promotes the expression of the miR-23a/24–2/27a cluster in MCF-7 cells, suggesting that c-Myc has an alternative functional role in a highly context-dependent manner in cell lines with different origins [25]. Whether c-Myc promotes or suppresses miR-23b and miR-27b at the transcriptional level in mouse neurons remains unknown.

In the present study, we observed that c-Myc expression was significantly higher in the mouse cerebral cortex and primary cortical neurons under hypoxia compared with that under normoxia. We found that c-Myc could directly downregulate the expression of miR-23b and miR-27b at the transcriptional level in mouse neurons. Furthermore, forced knockdown of c-Myc by siRNA could increase the transcription of miR-23b and miR-27b in neurons, whereas a c-Myc overexpression plasmid could inhibit the transcription of miR-23b and miR-27b in neurons. We further identified c-Myc binding sites in the promoter region of the miR-23b-27b cluster. Finally, we showed that elevated c-Myc expression led to an increased Apaf-1 level through suppressing miR-23b-27b expression, and this enhanced neuronal sensitivity to apoptosis. Taken together, these findings suggest that hypoxia could induce c-Myc expression to inhibit the transcription of miR-23b and miR-27b in neurons, resulting in the upregulation of Apaf-1.

## Materials and Methods

### Animals and treatment

C57BL/6 mice were employed in the present study. All animals were handled in accordance with the NIH Guidelines for the Care and Use of Laboratory Animals, and the protocols were approved by the Animal Care Committee of Nanjing University (Nanjing, China). Pregnant wild-type C57BL/6J mice were exposed to systemic hypoxia at the late stage of mouse gestation (gestation day 20; i.e., E19.5 for the pup). The mice were maintained in continuous hypoxia with an inspired O_2_ fraction of 6% for 6 h (gas mixture: 6% O_2_, 94% N_2_; hypoxic chambers, Coy Labs, Grass Lake, MI, USA). To enable adjustment to the hypoxic environment, the O_2_ levels were gradually decreased from an O_2_ fraction of 21% to 6% in 2% steps every 10 min, as described previously [26]. Controls were kept in the chamber under room air. The mother mice were sacrificed by cervical dislocation and brains were dissected out from embryos. Cerebral cortices were isolated, frozen in liquid nitrogen, and stored at -70°C until protein and RNA extraction.

### Cell culture, transfection and hypoxia treatment in vitro

Primary mouse cortical neuron cultures from E14.5-E15.5 C57BL/6 mice were obtained and maintained as previously described [27]. In brief, neocortices from fetal mice were dissociated, distributed on plates pre-coated with poly-D-Lysine (Sigma, St. Louis, MO, USA) and maintained in Neurobasal Medium (Gibco-Life Technologies, Grand Island, NY, USA) supplemented with 2% B27 (v/v, Gibco-Life Technologies), 1 mM glutamine, 100 IU/ml penicillin, and 100 mg/ml streptomycin. B27 minus AO (Gibco-Life Technologies) was substituted for B27 during the exposure of cortical neurons to hypoxia. Primary cortical neurons were transfected with plasmids or siRNAs at DIV3, as described previously [28]. For transfection, lipofectamine 3000 (Invitrogen, Carlsbad, CA, USA) and DNA or RNA were added Neurobasal medium individually and incubated at room temperature for 5 minutes. The two parts were then mixed and incubated at room temperature for another 25 minutes before adding onto cultured cortical neurons. Culture medium was replaced with conditioned medium (fresh medium mixed with 4-day-old culture medium at 1:1) the next morning. To induce hypoxia in cultured neurons, anaeroPack bags (Mitsubishi Gas Chemical Company, Inc., MGC, Japan) were employed as described [10].

### siRNA and c-Myc overexpression plasmid

The siRNAs targeting mouse c-Myc (sc-29227) and control siRNA (sc-37007) were purchased from Santa Cruz Biotechnology, Inc. The plasmid overexpressing Apaf-1 (pCMV6-c-Myc; MC216121) and the control plasmid (pCMV6; PS100020) were purchased from OriGene Technologies (Rockville, MD, USA).

### RNA isolation and Quantitative RT-PCR

Total RNA was extracted from tissues and cultured cells using Trizol reagent (Invitrogen) according to the manufacturer’s instructions. For quantitative RT-PCR analysis of mRNA, one μg of total RNA was reverse transcribed to cDNA with oligd(T) and Thermoscript (TaKaRa, Dalian, China). Real-time PCR was performed on an Applied Biosystems 7300 Sequence Detection System (Applied Biosystems, Foster City, CA, USA) using SYBR green dye (Roche, Mannheim, Germany). The sequences of the primers used for gene amplification were as follows: c-Myc (forward): 5’-GTGGTCTTTCCCTACCCG-3’; c-Myc (reverse): 5’-GTGTCCGCCTCTTGTCGT-3’; Apaf-1 (forward): 5’-GTTGATGCTGTCATTATGTAGGC-3’; Apaf-1 (reverse): 5’-AGGTAAAAGGGGAAGTATGTGTT-3’; GAPDH (forward): 5’-TGAAGCAGGCATCTGAGGG-3’; and GAPDH (reverse): 5’-CGAAGGTGGAAGAGTGGGAG-3’. Quantitative RT-PCR of mature miRNAs and pri-miRNAs was performed using TaqMan miRNA probes (Ambion, Austin, TX, USA) according to the manufacturer’s instructions. The ID number for mature miR-23b is 000400 and for mature miR-27b is 000409. The probes for pri-miRNAs were made to order and the ID number for pri-miR-23b is Mm03306184_pri and for pri-miR-27b is Mm03306468_pri. U6 snRNA or GAPDH mRNA were used to normalize the mature miRNA or pri-miRNA expression studies, respectively. A relative fold change in expression of the target gene transcript was calculated using the equation 2^-ΔCT^.

### Western blot analysis

Samples of tissues and cultured cells were lysed in RIPA sample buffer and centrifuged at 12000×g for 10 min at 4°C. The supernatant fraction was collected, and the protein concentration was quantified with a BCA assay (Pierce, Rockford, IL, USA). Proteins were separated on SDS-polyacrylamide gels and transferred to polyvinylidene difluoride membranes. The membranes were blocked for 1 h, followed by an overnight incubation at 4°C with primary antibodies. After washing, the membranes were incubated at room temperature for 1 h with the appropriate secondary antibody conjugated to horseradish peroxidase and then detected with an enhanced chemiluminescence reagent (Cell Signaling Technology Inc., USA). The intensity of each band was scanned and quantified using BandScan software (Glyko Inc., Novato, CA, USA). The following antibodies were used: anti-c-Myc (1:200, sc-40, Santa Cruz Biotechnology, Inc.) and anti-Apaf-1 (1:500, ab32372, Abcam, Cambridge, MA, USA).

### Chromatin immunoprecipitation (ChIP)

ChIP assays were carried out using an EZ-ChIP assay kit (Upstate Biotechnology, Inc.) in accordance with the manufacturer’s instructions. Soluble chromatin was prepared from primary cortical neurons and incubated with anti-c-Myc antibody (sc-40x, Santa Cruz Biotechnology, Inc.) or mouse IgG as a negative control. The primer pairs used for PCR analysis are as follows:

for site 1:

5’-GCAGTGGGTGCCTGTAAG-3’ (forward) and 5’-CTGTTTGGCTCCGTTTCG-3’ (reverse);

for site 2:

5’-GGGACTTGATGAAATGAGC-3’ (forward) and 5’-TGAACTTAACTGTGGGTTG-3’ (reverse);

and for site 3:

5’-CACTGGGTAGCAGTTGGA-3’ (forward) and 5’-ACAGCAGGGTGTTAGAAG-3’ (reverse). All data were normalized to the input.

### Luciferase reporter constructs and luciferase assay

Promoter of miR-23b-27b cluster was amplified by PCR from mouse genomic DNA. PCR primers were listed as follows: 5′-CGACGCGTCGGCAAACAGGCAGAAGCAC-3′ (forward) and 5′-CCGCTCGAGCGGCAACCACTCCCATCCACA-3′ (reverse). The PCR products were separated by agarose gel electrophoresis, and the DNA fragments then isolated and cloned into the restriction enzyme (MluI and XhoI) digested pGL3 Basic Vector (Promega, Madison, WI, USA). The construct was confirmed by sequencing. Site 1 was mutated from CACGTG to CTCGAG. HEK-293T cells were cultured in 24-well plates, and each well was transfected with luciferase reporter plasmid, β-galactosidase (β-gal) expression vector (Ambion), and c-Myc overexpression vector using the lipofectamine 3000 reagent (Invitrogen) according to the manufacturer’s instructions. The b-gal vector was used as a transfection control. At 24 h post transfection, the cells were assayed using luciferase assay kits (Promega). The data depicted are representative of three independent experiments performed on different days.

### Immunofluorescence staining

Primary cortical neurons after hypoxia treatment were fixed in 4% paraformaldehyde, washed with PBS for 30 min and blocked for 1 h at room temperature. The samples were then incubated with primary antibody at 4°C overnight, followed by incubation with an Alexa Fluor 594-conjugated secondary antibody (1:1000, Invitrogen) in a dark room at room temperature. Nuclei were counterstained with DAPI (Sigma) for 5 min and then washed in PBS.

### TUNEL Assay

Briefly, primary cortical neurons were transfected with c-Myc siRNAs at DIV3 followed by hypoxia treatment at DIV5. The cells were harvested, washed and fixed with 4% paraformaldehyde. TUNEL assays were performed with an in situ apoptosis detection kit (KeyGEN BioTECH, Nanjing, China) according to the manufacturer’s instructions.

### Statistical analysis

The data are presented as the mean ± S.E.M. of at least three independent experiments. Direct comparisons were made using Student’s t-tests, and multiple group comparisons were made using one-way analyses of variance (ANOVA). Statistical significance was defined as P < 0.05, 0.01, or 0.001 (indicated as *, **, or ***, respectively). P-values ≥0.05 were considered not significant (indicated as NS). Prism 5.0 software (GraphPad, Inc., La Jolla, CA, USA) was used for data analyses.

## Results

### Hypoxia causes increased c-Myc protein expression and decreased miR-23b-27b in mouse cerebral cortex and primary cortical neurons

In our previous study, we showed that the levels of miR-23a/b and miR-27a/b in the cortices of E19.5 mice were decreased during hypoxia [10]. However, the mechanism by which the miRNA expression is regulated in neurons remains unknown, and the pathway by which hypoxia downregulates the expression of miR-23a/b and miR-27a/b is also unknown.

**Fig 1:**
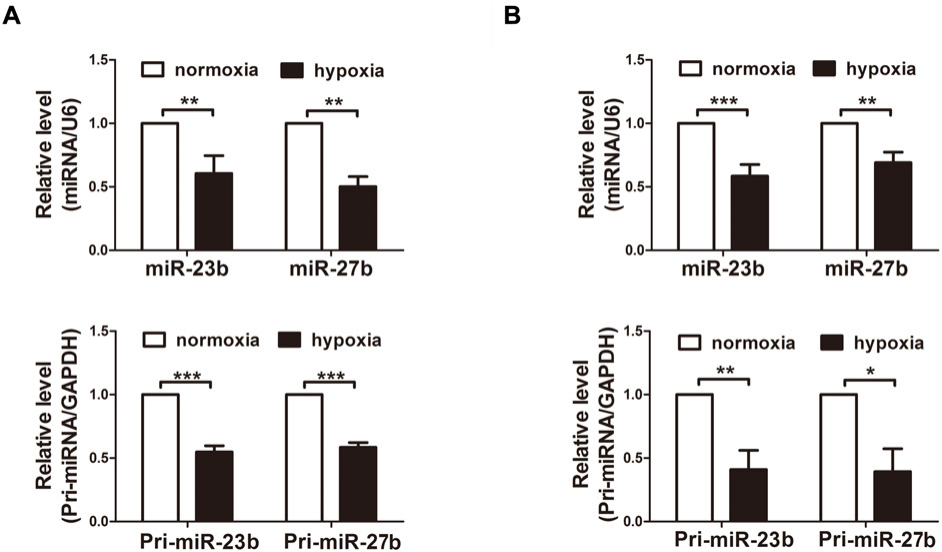
Hypoxia treatments decrease the levels of mature miR-23b and miR-27b and their primary transcripts. A and B, Quantitative RT-PCR detection of the expression of mature and primary form of miR-23b and miR-27b in the cerebral cortices (A) and in primary cultures of cortical neurons (B) under conditions of normoxia or hypoxia for 6 h (n = 4, unpaired t-test, * P < 0.05, ** P < 0.01, *** P<0.001).

To determine whether the miRNAs are regulated at the transcriptional level, we simultaneously examined the expression levels of both mature miR-23b/27b and their primary transcripts. We observed that the expression levels of pri-miR-23b/27b were significantly lower in the mouse cortex and primary cortical neurons after hypoxia treatment. The similar expression patterns of the pri-miRNA and mature miRNA indicate that the transcription of the miR-23b-27b cluster is downregulated during hypoxia (Fig. 1A-B).

**Fig 2:**
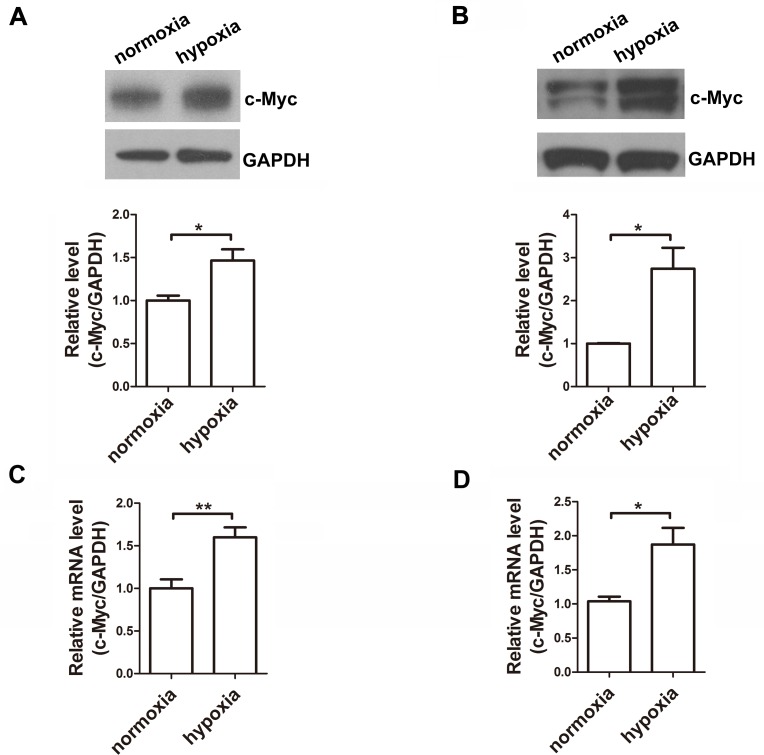
c-Myc level is elevated in both mRNA and protein levels in cortices of E19.5 pups under hypoxia. A and B, Representative western blot images for c-Myc in the cerebral cortices (A) and in the primary cortical neurons (B) under conditions of normoxia or hypoxia for 6 h. Relative fold changes in the c-Myc protein level were quantified by densitometry (n = 4, unpaired t-test, * P < 0.05). C and D, Quantitative RT-PCR detection of c-Myc mRNA expression in the cerebral cortices (C) and in the primary cortical neurons (D) under conditions of normoxia or hypoxia for 6 h (n = 4, unpaired t-test, * P < 0.05, ** P < 0.01).

Previous studies have reported that c-Myc suppresses miR-23a/b [24] or promotes the miR-23a/24–2/27a cluster at the transcriptional level [25] in different cell lines. It has been reported that c-Myc is increased in the turtle brain after hypoxia [22]. We therefore examined the possible role of c-Myc in regulating miR-23b and miR-27b in our model. Both of the protein and mRNA expression of c-Myc were increased in the mouse cortex and primary cortical neurons after hypoxia treatment (Fig. 2A-D). Therefore, the inversely correlated expression patterns of miR-23b-27b cluster and the c-Myc gene in this model indicate that c-Myc might suppress miR-23b and 27b expression in neurons during hypoxia.

### c-Myc downregulates miR-23b and miR-27b at the transcriptional level

**Fig 3:**
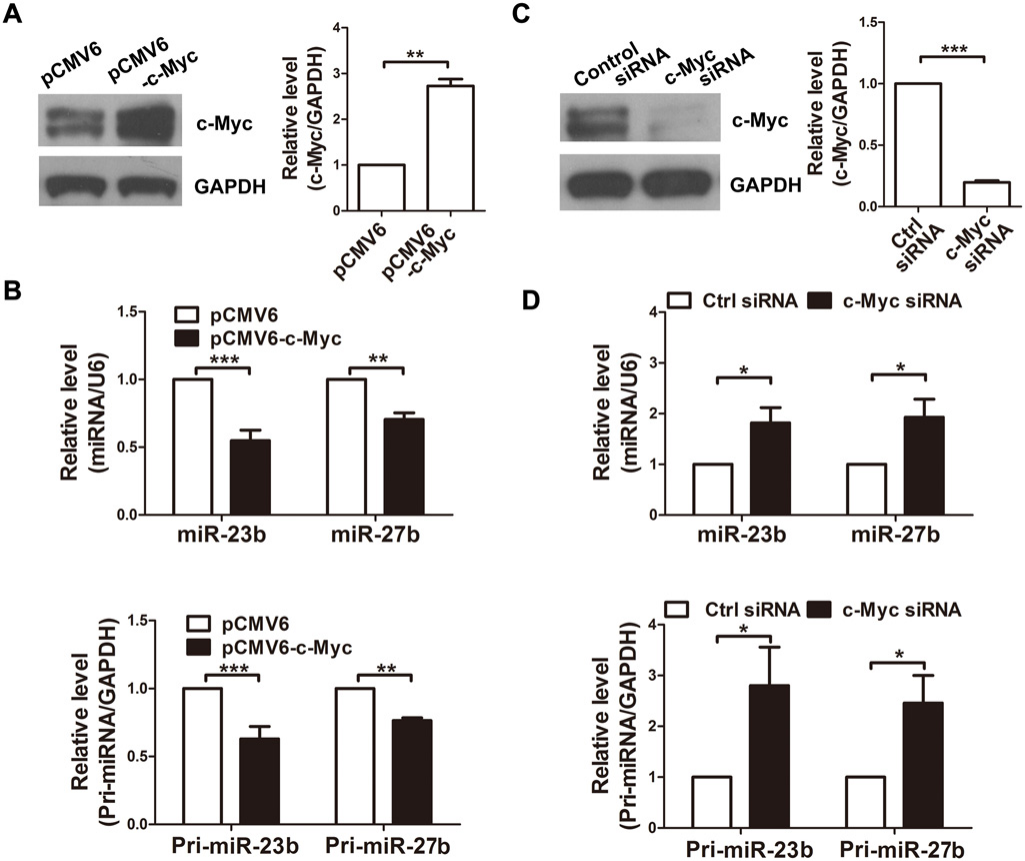
c-Myc downregulates miR-23b and miR-27b expression in cultured neurons at the transcriptional level. A, Western blot analysis of c-Myc in primary cortical neurons 48 h after transfection with plasmids (pCMV6 or pCMV6-c-Myc). Relative amounts of the c-Myc protein were quantified by densitometry (n = 4, unpaired t-test, ** P<0.01). B, Quantitative RT-PCR detection of the expression of mature and primary form of miR-23b and miR-27b in primary cortical neurons pre-transfected with plasmids (n = 4, unpaired t-test, ** P < 0.01, and *** P<0.001). C, Western blot analysis of c-Myc in primary cortical neurons 48 h after transfection with c-Myc siRNA. Relative amounts of c-Myc protein were quantified by densitometry (n = 3, unpaired t-test, *** P<0.001). D, Quantitative RT-PCR detection of the expression of mature and primary form of miR-23b and miR-27b in primary cortical neurons pre-transfected with siRNA (n = 4, unpaired t-test, * P < 0.05).

To further determine whether c-Myc could inhibit miR-23b and miR-27b expression in neurons, we placed a c-Myc expression vector into the neurons to elevate the c-Myc protein level (Fig. 3A). Real-time PCR analysis showed that mature the expression of mature and primary form of miR-23b and miR-27b were significantly decreased (Fig. 3B). On the contrary, a specific siRNA of c-Myc was employed to knock down c-Myc expression (Fig. 3C). We found that the expression of mature and primary form of miR-23b and miR-27b were significantly increased above control levels (Fig. 3D). Together, these findings demonstrate that c-Myc negatively regulates the expression of miR-23b and miR-27b in primary cortical neurons.

### c-Myc acts at an E-Box to repress miR-23b and miR-27b expression

**Fig 4:**
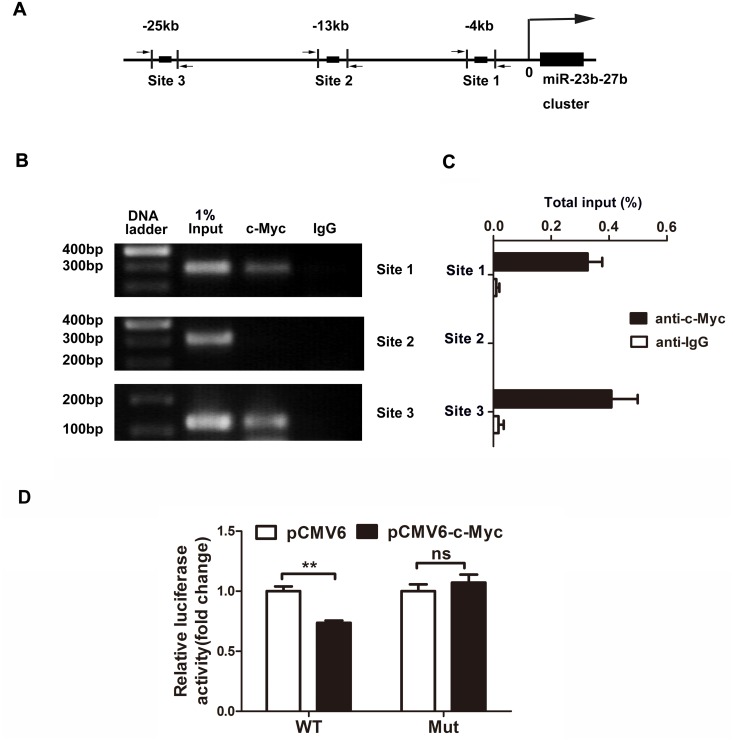
c-Myc acts on E-Boxes in the promoter of the miR-23b-27b cluster. A, Diagram representing the c-Myc binding sites in the miR-23b-27b cluster promoter region. B and C, ChIP assays were performed by IP with either anti-c-Myc antibody or control IgG. D, c-Myc regulates the promoter of the miR-23b-27b cluster. For the luciferase reporter assay, cells were co-transfected with empty vector or c-Myc plasmids and the luciferase reporter plasmid carrying promoter constructs containing site 1 or site 1 mutant (n = 3, unpaired t-test, ns, not significant,** P < 0.01).

Because we demonstrated that c-Myc suppressed the expression of miR-23b and miR-27b, we next tested whether c-Myc could directly bind to the promoter region of miR-23b-27b cluster. We searched for the DNA response elements (the binding sites for c-Myc) in the genomic region containing the miRNA promoter. Approximately thirty kilobases of DNA sequence on chromosome 13 upstream of the mir-23b-27b cluster was analyzed for putative c-Myc-binding sites. c-Myc is known to bind to the canonical E-box sequence CACGTG, and we identified three putative binding sites matching sequence at 4 kb, 13 kb and 25 kb upstream from the transcriptional starting site (Fig. 4A).

To test whether c-Myc could bind to the promoter of miR-23b-27b cluster, we performed ChIP assays. We observed a significant enrichment of the miR-23b-27b cluster promoter amplicon in c-Myc ChIP samples of site 1 and site 3 compared with ChIP samples generated with control IgG (Fig. 4B-C). There was no c-Myc binding signal on site 2. This result clearly showed that c-Myc binds directly to the miR-23b-27b cluster locus, providing strong evidence that these miRNAs are directly regulated by this transcription factor.

We also generated luciferase reporter constructs covering the potential element-site1, as well as mutation, of the putative promoter region of miR-23b-27b cluster to test the transcriptional regulation by c-Myc. As shown in Fig. 4D, overexpressing c-Myc suppressed the luciferase activity in cells transfected with the luciferase constructs that encompass the putative c-Myc binding site at-4 kb. Conversely, after introducing mutations to predicted binding site “CACGTG”, the inhibitory effect of overexpressing c-Myc was abolished. This result indicates that c-Myc transcriptionally represses miR-23b-27b cluster by binding to the upstream canonical E-box element of miR-23b-27b cluster.

### Knockdown of c-Myc suppresses Apaf-1 through elevating the expression of miR-23b-27b cluster and attenuates neuronal apoptosis induced by hypoxia

**Fig 5:**
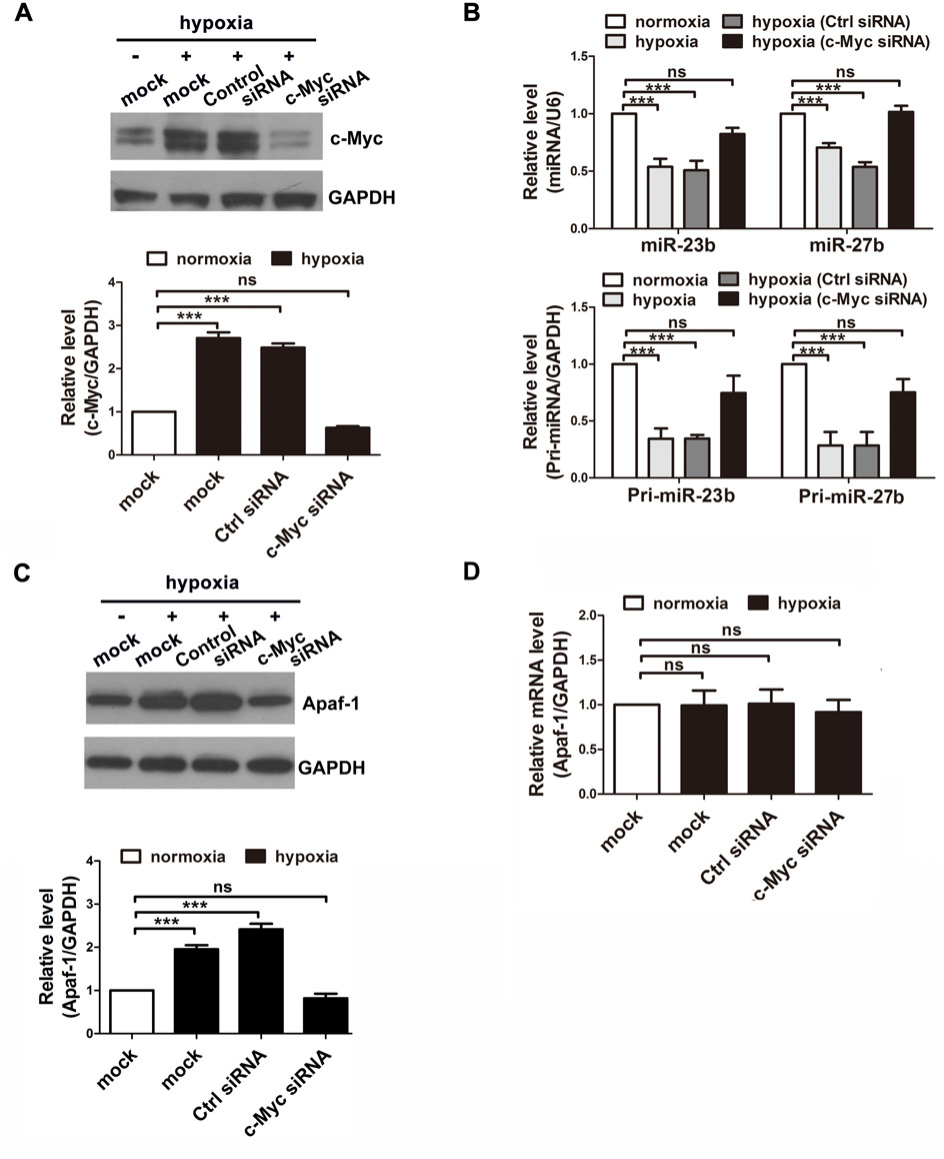
Knockdown of c-Myc recovers the miR-23b and miR-27b levels decreased by hypoxia. A, Representative western blot images for c-Myc in the primary cortical neurons pre-transfected with control or c-Myc siRNAs under conditions of normoxia or hypoxia for 6 h. The relative amounts of the c-Myc protein were quantified by densitometry (n = 3, one-way ANOVA with Newman-Keuls multiple comparison test, ns, not significant, *** P<0.001). B, Quantitative RT-PCR detection of the expression of mature and primary form of miR-23b and miR-27b in primary cortical neurons pre-transfected with siRNA under conditions of normoxia or hypoxia for 6 h (n = 5, one-way ANOVA with Newman-Keuls multiple comparison test, ns, not significant, *** P<0.001). C and D, Representative western blot images for Apaf-1 (C) and quantitative RT-PCR detection of Apaf-1 mRNA expression (D) in the primary cortical neurons pre-transfected with control or c-Myc siRNAs under conditions of normoxia or hypoxia for 24 h. Relative amounts of Apaf-1 protein were quantified by densitometry (n = 3, one-way ANOVA with Newman-Keuls multiple comparison test, ns, not significant, *** P<0.001).

We further sought to investigate the role of c-Myc in neuronal apoptosis induced by hypoxia. Cortical neurons were transfected with the c-Myc siRNA; 48 h later, the neurons received anaerobic treatment (6 h), and we found that knocking down c-Myc expression restored the miR-23b and miR-27b levels (Fig. 5A-B).

**Fig 6:**
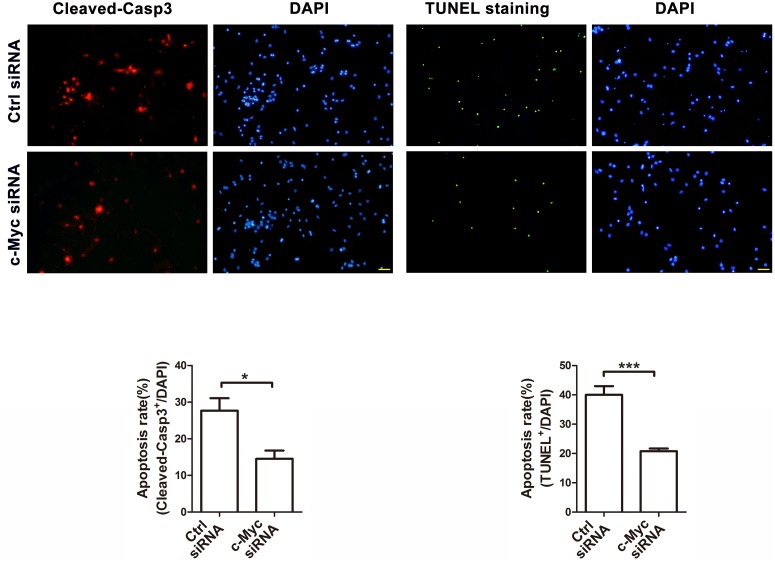
Knockdown of c-Myc attenuates neuronal apoptosis induced by hypoxia. Immunofluorescence staining of neuronal apoptosis. Neurons pre-transfected with control or c-Myc siRNAs were stained with cleaved caspase-3 antibody or with a TUNEL staining kit after hypoxia treatment (24 h) at DIV5. Scale bar represents 50μm. Statistical analysis of the percentage of cleaved caspase-3-positive neurons or TUNEL-positive neurons to DAPI-stained cells (cell numbers = 500–600, unpaired t-test, * P < 0.05, *** P<0.001).

To further determine whether the recovered miR-23b and miR-27b expression could regulate the targeting protein Apaf-1, we assessed the protein level of Apaf-1 in neurons treated with c-Myc siRNA and subjected to anaerobic treatment (24 h). We found that Apaf-1 was elevated at this time point [10]. As shown in Fig. 5C, the increased Apaf-1 protein expression caused by hypoxia was reduced to the control level by knockdown of c-Myc. While, the mRNA expression of Apaf-1 was unchanged (Fig. 5D), which indicated that Apaf-1 was regulated at the post-transcriptional level by miRNA. Importantly, we found that the neuronal apoptosis rate, calculated as the cleaved caspase-3-positive neurons or TUNEL-positive neurons, was significantly decreased in the siRNA group (Fig. 6). In conclusion, these results indicate that knockdown of c-Myc alleviates neuronal apoptosis by enhancing miR-23b and miR-27b and inhibiting Apaf-1 in hypoxia-induced pathological conditions.

## Discussion

We have reported that mature miR-23b and miR-27b are downregulated during hypoxia [10]. In the present study, we observed that the expression levels of the primary transcripts of miR-23b and miR-27b were also significantly lower in the hypoxia group, suggesting that miR-23b and miR-27b may be regulated by transcription factors. We found that c-Myc could repress miR-23b and miR-27b at the transcriptional level in primary cortical neurons. We also found that hypoxia could increase the expression of c-Myc. The elevated c-Myc level suppressed the expression of miR-23b and miR-27b, and resulted in higher Apaf-1 levels and enhanced sensitivity to apoptosis.

As a transcription factor, c-Myc participates in controlling the expression of many target genes and forms a complicated network with other transcription factors. Many studies reveal that c-Myc suppresses miRNA expression and contributes to tumorigenesis [24,29,30]. Previous studies have shown that c-Myc differentially regulates miR-23 and miR-27 expression at the transcriptional level in different cell types [24,25]. Consistent with Gao’s result [24], we found that c-Myc suppressed miR-23b and miR-27b expression, and we identified two binding sites in the miR-23b-27b cluster in primary cultured neurons. Interestingly, Xue et al also reported that miR-15–16 is repressed and that there are three c-Myc binding sites in the promoter of miR-15–16 during hypoxia in colorectal carcinoma cell lines [30]. These studies indicate that c-Myc might be an inhibitory transcription factor for miRNA expression during hypoxia. The detailed mechanism by which c-Myc regulates miRNAs requires further investigation.

It has been shown that systemic hypoxia at the very end of pregnancy induces simultaneous upregulation of the major hypoxia-inducible transcription factors (HIFs) HIF-1 and HIF-2 in the developing mouse brain and placenta, implying that placental HIFs represent markers of fetal cerebral hypoxic distress [26]. HIF is an α/β heterodimer. HIF-1α and HIF-2α play critical roles in the cellular response to hypoxia [31,32]. HIF-2α expression is transcriptionally increased by acute and chronic hypoxia, whereas HIF-1α mRNA levels are either decreased or largely unchanged under the same conditions [31]. Moreover, HIF-2α appears to be preferentially expressed in neuronal tumor cells with cancer stem cell characteristics [33,34], and it promotes hypoxic cell proliferation by enhancing c-Myc transcriptional activity [35]. The c-Myc-mediated transcriptional repression of miR-15–16 in hypoxia is induced by increased HIF-2α [30]. These results suggest that HIF-2α may play a more vital role in the function of neuronal cells under hypoxia. Thus, the elevated c-Myc expression in neurons under hypoxia may be attributed to the expression change of HIF-2α.

Our previous study showed that miR-23/27 regulates neuronal sensitivity to apoptosis by suppressing Apaf-1 expression [10]. Apaf-1 has been identified as a key functional protein in physiological apoptosis during brain development and in pathological apoptosis related to CNS injury [36–38]. As a key apoptotic protein that may decide the apoptotic fate of cells, Apaf-1 expression is tightly regulated. We show that c-Myc could suppress the transcription of pri-miR-23b/27b, resulting in a lower level of mature miR-23b/27b. The decreased miR-23b/27b expression led to greater expression of their target, the Apaf-1 protein, which enhanced the neuronal cells’ sensitivity to hypoxia-induced apoptosis.

In conclusion, our findings suggest a potential molecular mechanism underlying the expression change of miRNAs during fetal distress. The acute brain injury induced by hypoxia appears to promote the expression of c-Myc and suppress the expression of miR-23b and miR-27b, consequently leading to greater neuronal apoptosis. Downregulating the expression of c-Myc may help to protect neurons from injury-induced apoptotic cell death during hypoxia, and according to our results, c-Myc may serve as a novel therapeutic target in hypoxia-induced neuronal apoptosis during fetal distress.
